# Experimental ectoparasite removal has a sex‐specific effect on nestling telomere length

**DOI:** 10.1002/ece3.9861

**Published:** 2023-03-07

**Authors:** Sarah E. Wolf, Samuel Zhang, Ethan D. Clotfelter

**Affiliations:** ^1^ Department of Biology Indiana University Bloomington Indiana USA; ^2^ Department of Biobehavioral Health Pennsylvania State University State College Pennsylvania USA; ^3^ Department of Biology Amherst College Amherst Massachusetts USA

**Keywords:** blood, ectoparasite, growth, permethrin, sex, telomere length

## Abstract

Parasites are a strong selective force that can influence fitness‐related traits. The length of chromosome‐capping telomeres can be used to assess the long‐term costs of parasitism, as telomere loss accelerates in response to environmental stressors and often precedes poorer survival prospects. Here, we explored the sex‐specific effects of ectoparasite removal on morphology and telomere length in nestling tree swallows (*Tachycineta bicolor*). To do so, we experimentally removed blow fly (*Protocalliphora* spp.) larvae from nests using Permethrin, a broad‐spectrum insecticide. Compared to water‐treated controls, insecticide treatment of nests had a sex‐biased effect on blood telomere length: ectoparasite removal resulted in significantly longer telomeres in males but not females. While this treatment did not influence nestling body mass, it was associated with reduced feather development regardless of sex. This may reflect a relaxed pressure to fledge quickly in the absence of parasites, or alternatively, could be a negative side effect of permethrin on morphology. Exploring robust sex‐specific telomere dynamics in response to early‐life environmental pressures such as parasitism will shed light on sexual dimorphism in adult life histories and aging.

## Graphical abstract

Experimental removal of ectoparasites from the nests of tree swallows led to longer telomeres in male, but not female nestlings by 12 days old. This suggests that male physiology may be more sensitive to parasitism in this species, which could contribute to sex variation in life history and aging.

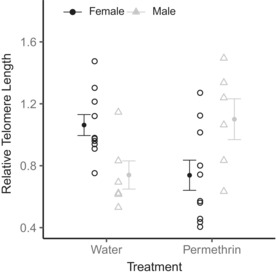

## 1 INTRODUCTION

Parasites are a taxonomically diverse group of organisms that often prompt trade‐offs in their hosts and influence fitness (Bower et al., 2019; Lehmann, 1993). Work across a broad taxonomic range of animals shows that parasites can reduce host survival, reproductive output, and offspring quality (Møller et al., 1990; Richner et al., 1993; Schwanz, 2008; Watson, 2013). At broader scales, parasites also regulate host populations and limit population viability (McCallum et al., 2009; Poulin, 2007; Smith et al., 2009; Tompkins et al., 2011). A better understanding of such selective outcomes requires a close examination of the proximate mechanisms underlying consequences of parasitism at the organismal level.

One potential mechanism by which parasites may reduce individual host fitness is via reduction in telomere length. Telomeres are protective caps of chromosomes that shorten during cellular replication (Blackburn, 2005). Telomere loss is often accelerated with environmental stressors, including infection status (Chatelain et al., 2020). The accumulation of short telomeres—which are thought to signal lower quality DNA and changes in gene expression (Baur et al., 2001; Kawanishi & Oikawa, 2004), often predicts poorer survival across vertebrates (Wang et al., 2018; Whittemore et al., 2019; Wilbourn et al., 2018). Work on human subjects has previously tied chronic infection to telomere loss (Bellon & Nicot, 2017; Effros, 2011), a pattern corroborated by animal studies on endo‐ and ectoparasites in the wild (Asghar et al., 2015, 2016; Karell et al., 2017; Soler et al., 2017). For example, Asghar et al. (2016) experimentally infected captive siskins (*Spinus spinus*) with endoparasitic malaria (*Plasmodium* spp.) and found that telomere loss was correlated with an individual's infection intensity. However, sparse studies make it unclear whether additional factors (e.g., taxonomic pairings, and host sex) mediate the mechanistic links between parasitism and telomeres.

Parasitism may alter telomeres via changes to oxidative status. First, parasites can activate energetically expensive immune responses (Demas et al., 1997; Martin et al., 2003; Saino et al., 1998; Svensson et al., 1998), which may increase glucocorticoids and oxidative stress (Athanasoulia‐Kaspar et al., 2018; Costantini, 2022; O'Dwyer et al., 2020; von Zglinicki, 2002; but see Reichert & Stier, 2017). Imperfect repair of DNA damage induced by oxidative stress could then shorten telomeres. In addition, parasites can stunt body growth (Hoi et al., 2018; as seen in birds: Pryor & Casto, 2017; Romano et al., 2021; but see Dugas & Border, 2022a) in multiple ways. Parasites may limit resource allocation (Lochmiller & Deerenberg, 2000) toward body growth via consumption of blood or by increasing metabolic rates during infection (Cutrera et al., 2022; Lind et al., 2020; Ots et al., 2001; but see Smith et al., 2017). Ectoparasitism in particular may also cost time and energy via increased grooming (Mooring et al., 1996; Simon et al., 2005), although this removal of parasites could aid host nutrition (Johnson et al., 2010). Interestingly, parasitism is also linked to low quality ornaments in young birds, which may influence resource allocation among siblings from parents (Dugas & Border, 2022a, 2022b; Romano et al., 2021). Such stunted growth could theoretically decrease oxidative stress and slow telomere loss (Alonzo‐Alvarez et al., 2007; Monaghan & Ozanne, 2018; but see Vedder et al., 2017); however, slowed growth accompanied by other parasite‐induced oxidative stress may still lead to telomere loss. Despite these generalities, studies of parasites produce inconsistent effects on physiology, which may be mediated by internal and environmental factors.

Host biological sex is one factor that may alter effects of parasitism. Among mammals, males often appear more intensely parasitized than females (Moore & Wilson, 2002; Stephenson et al., 2016; Waterman et al., 2014; but see O'Brien & Dawson, 2013). Male‐biased parasitism may be strong in species with larger or more ornamented males (Christe et al., 2003; Hawlena et al., 2005), as these individuals may be larger targets with greater resource availability for parasites (Rosso et al., 2020). However, the smallest individuals with the poorest immunity may also be targeted, although support is mixed in birds (Christe et al., 1998; Garrido‐Bautista et al., 2022; Roulin et al., 2003). In addition, adult males may perform riskier mating‐related behaviors that increase exposure to infection (Habig et al., 2018). Once infected, immunosuppression via higher testosterone may heighten male infection intensity, although results are mixed (Foo et al., 2017; Roberts et al., 2004). Consequently, infections of mice with *Salmonella enterica* shorten male telomeres (Ilmonen et al., 2008). Similarly, more virulent haemosporidian species are associated with telomere loss in male, but not female, blue tits (*Cyanistes caeruleus*; Sudyka et al., 2019). However, Tschirren et al. (2021) showed that exposure to ectoparasitic hen fleas (*Ceratophyllus gallinae*) causes shorter telomeres in female nestling great tits (*Parus major*) without effects on males. Altogether, this highlights a current lack of consensus among studies on the directionality of sex‐biased telomere loss following infection.

We tested the hypothesis that ectoparasitism shortens telomere length in a sex‐specific manner in nestling tree swallows (*Tachycineta bicolor*). To do so, we used the broad‐spectrum insecticide Permethrin to experimentally remove *Protocalliphora* blow flies from the nesting material. Nestling birds are particularly vulnerable to parasites because their behavioral (e.g., preening) and physiological defenses are not yet fully developed (Aastrup & Hegemann, 2021; Koop et al., 2013). Furthermore, telomere loss is typically fastest early in life (Monaghan, 2010) and can predict adult body size, survival, and reproductive success (Caprioli et al., 2013; Eastwood et al., 2019; Heidinger et al., 2012); therefore, parasite‐induced variation in telomere dynamics at this age may play a critical role in adult success. Given evidence that males are more susceptible to infections, we expect that parasite removal will result in male‐biased increases in body size and telomere length.

## 2 MATERIALS AND METHODS

### 2.1 Study system

Tree swallows were studied near Amherst, MA USA (42.361°N, 72.509°W, elevation 50–60 m asl) in May–July 2020. Our study area includes 120 nest boxes, approximately two‐thirds of which were occupied by tree swallows. Boxes were situated 20–30 m apart from each other and oriented in random directions. We monitored each active nest box to determine the day on which the first egg hatched (day 0).

### 2.2 Experimental manipulation of ectoparasites

**FIGURE 1:**
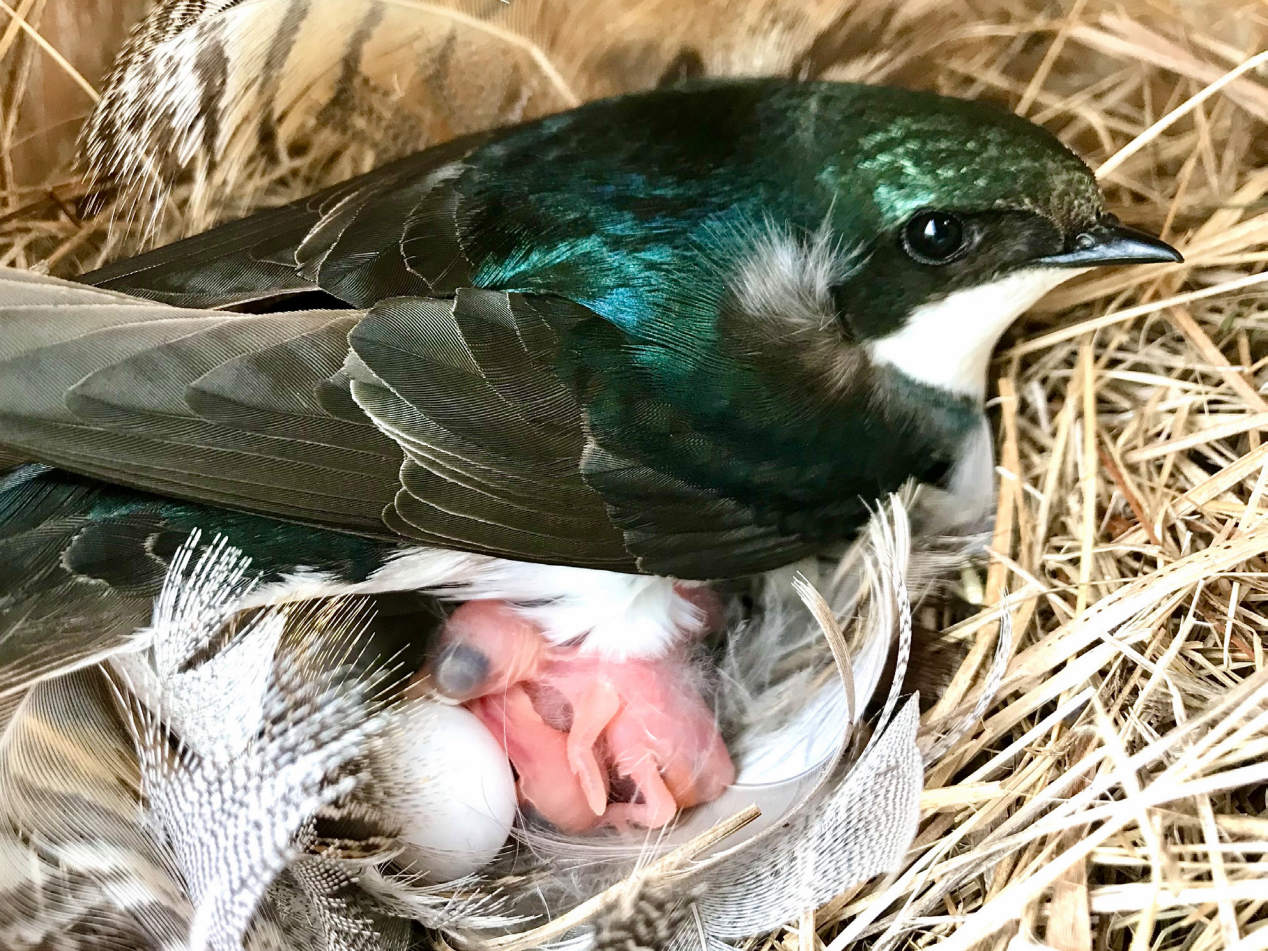
A female tree swallow (*Tachycineta bicolor*) warming newly hatched nestlings and one remaining egg on a typical nest made of dry grass and feathers, which can often house ectoparasites, e.g. blow flies (*Protocalliphora* spp.). Image credits: Samuel Zhang (author), Amherst College.

Tree swallows are obligate secondary cavity nesters that host a wide variety of ectoparasites, including mites, lice, fleas, and flies (Figure 1). These ectoparasites feed on blood, skin, and feathers (Janovy et al., 1997; Rendell & Verbeek, 1996) and can negatively impact offspring physiology, immune function, and survival (López‐Arrabé et al., 2015; Martínez‐de La Puente et al., 2011; Merino & Potti, 1995; Saino et al., 1998). Blow flies (*Protocalliphora*) have been found to infest 65.9% of tree swallow nests in northeastern US (Roby et al., 1992), with loads ranging from 4 to 54 parasites per nest (Grab et al., 2019). Previous work shows that blow flies feed on nestling blood and can cause anemia, hyperglycemia, and increased metabolic rates in avian hosts (De Simone et al., 2018; Grab et al., 2019; Pryor & Casto, 2015; Sun et al., 2020). One broad‐spectrum insecticide commonly used to remove ectoparasites is permethrin, which attacks the nervous system of larval and adult insects (Edwards, 2006). Permethrin treatment is effective against blow flies in nests of tree swallows (De Simone et al., 2018; Grab et al., 2019) and other bird species (Bulgarella et al., 2020) and also decreases the abundance of other ectoparasites like fleas and mites (Harriman et al., 2014; Pap et al., 2005; Pryor & Casto, 2017).

Any nest with a known hatching day was selected for our experiment, and then randomly assigned to one of two treatment groups, one in which ectoparasites were eliminated via the application of the insecticide permethrin (Permectrin II ©, diluted to 1% with distilled water), the other in which nests were treated with water as a control. In both treatment groups, nests were sprayed on day 0 and again on day 4. To do this, nestlings were temporarily removed from the nest, the bottom and sides of the nest were sprayed thoroughly (to minimize direct contact with nestlings), and the nestlings were returned once the nest had completely dried approximately 5 min later. To the extent possible, permethrin‐ and water‐treated nests were paired by hatch date to avoid the confounding effects that date has on many aspects of tree swallow reproduction (Winkler et al., 2020). The final number of nests in our study (*n* = 16 control, *n* = 16 insecticide) was less than the initial number sprayed due to brood loss.

### 2.3 Nestling measurements

On day 11, we returned to nest boxes to collect morphological data and a blood sample. We measured body mass (±0.01 g), wing chord length (±1 mm), and the amount of seventh primary feather emergence on the right wing (±0.1 mm), which is strongly correlated with ninth (outermost) primary feather emergence (personal observation: *R* ^2^ = 0.90; *F*_1,189_ = 725.78, *p* < .0001). Seventh primary emergence is often greater than ninth primary emergence at this age, making it a slightly more precise measurement. Body mass is thought to reflect an individual's overall quality and has been linked to post‐fledging survival (e.g., McCarty, 2001). On the other hand, wing length and feather emergence strongly predict flight ability (Jones et al., 2020) and post‐fledging predation (Jones et al., 2017), although we suspect wing measurements are also important for food acquisition for these aerial insectivores (Winkler et al., 2020).

Blood was collected from one nestling in each nest. In order to isolate the effects of ectoparasite presence and sex on telomere length and morphology, we sampled nestlings semi‐randomly to avoid those of extremely low or high body mass, i.e., we selected those who were similarly‐sized targets for blow flies and did not experience atypical growth rates. We also avoided collecting blood from runts because these bleeds can be unreliable and may decrease the probability of successful fledging. We punctured the brachial vein with a 26 g needle and collected 75–150 μL of blood in heparinized microhematocrit capillary tubes. Blood samples were transferred to 0.5 mL microcentrifuge tubes on ice while in the field and were then stored at −80°C.

### 2.4 Quantifying ectoparasites

We assessed the efficacy of permethrin by estimating blow fly (*Protocalliphora sialia*) load in each control and treated nest. Following past work (De Simone et al., 2018; Grab et al., 2019), we collected all nests in sealed bags after fledging (19–21 days post‐hatch). Nests were then dissected in the laboratory, by one observer (SZ) blind to the experimental treatment group, to count blow fly larvae and pupae. Permethrin effectively reduced ectoparasite load (zero‐inflated negative binomial regression: *p* = .003), where blow flies were found in 0% of permethrin‐treated nests and 43.8% of water‐treated nests (9.5 ± 4.27 per nest across all control nests; 21.71 ± 7.76 per nest across control nests with blow flies; minimum = 1; maximum = 54). Note that feather mites and lice were likely present in small numbers but were not quantified, although we did note several random observations of lice on nestlings. Therefore, any effects of permethrin on morphology and telomere length can only be attributed to blow fly reduction, although it is possible that removal of other ectoparasites also contributed. In addition, our estimates do not distinguish sibling variation in ectoparasite exposure and therefore, only estimates a nest‐average exposure for our nestlings of interest.

### 2.5 Molecular sexing

We sexed all nestlings using the P2/P8 method developed by Griffiths et al. (1998) and validated in Wolf et al. (2022), which amplifies two conserved CHD genes on the avian sex chromosomes. Each reaction contained 5 μL PerfeCTA SYBR Green SuperMix No Rox (Quanta Biosciences), 0.2 μL 200 nM P2 primer (5′‐TCT‐GCA‐TCG‐CTA‐AAT‐CCT‐TT‐3′), 0.2 μL 200 nM P8 primer (5′‐CTC‐CCA‐AGG‐ATG‐AGR‐AAY‐TG‐3′), 3.6 μL dH_2_O, and 1 μL extracted DNA (range: 100–300 ng/μL). We ran PCR under the following conditions: (95°/2 m) + 40× (95°/30 s, 51°/45 s, 72°/45 s) + 72°/5 m + 4° hold. PCR products were run on a 3% EtBr agarose gel (1.5 h at 90 V) alongside known adult male and female samples. Males exhibited a single band at ~250 bp and females exhibited a double band at ~250 and 275 bp. Sex ratios in each treatment group were as follows: *n* = 6 male and 10 female controls; *n* = 6 male and 10 female experimentals.

### 2.6 Quantification of telomere length

We quantified relative telomere length using qPCR (adapted from Cawthon, 2009; Criscuolo et al., 2009), which used DNA extracted from ≤25 μL whole blood via an automated Maxwell® RSC Instrument (Promega, Madison, WI) and Whole Blood DNA Kit (Promega no. AS1520). Relative telomere length was measured as the ratio (T/S) of telomere repeat copy number (T) to a single gene copy number (S), relative to a pooled reference sample. We amplified our single copy gene, glyceraldehyde‐3‐phosphate dehydrogenase (GAPDH) using primers GAPDH‐F (5′‐AAC‐CAG‐CAA‐AGT‐ACG‐ATG‐ACA‐T‐3′) and GAPDH‐R (5′‐CCA‐TCA‐GCA‐GCA‐GCC‐TTC‐A‐3′). We amplified telomeres using primers telg (5′‐ACA‐CTA‐AGG‐TTT‐GGG‐TTT‐GGG‐TTT‐GGG‐TTT‐GGG‐TTA‐GTG‐T‐3′) and telc (5′‐ TGT‐TAG‐GTA‐TCC‐CTA‐TCC‐CTA‐TCC‐CTA‐TCC‐CTA‐TCC‐CTA‐ACA‐3′). We quantified all Ct values for GAPDH and telomeres on a single 384‐well plate (ABI Quantstudio 5, Foster City, CA). Prior to plating, we diluted DNA samples to 3.33 ng/μL using ultrapure water. Each reaction had a total volume of 10 μL containing 5 μL PerfeCTA SYBR Green SuperMix Low ROX (Quanta Biosciences, Gaithersburg, MD, USA), 200 nM each GAPDH‐F/GAPDH‐R or 200 nM each telc/telg, and 3 μL DNA extract (10 ng total). qPCR reaction conditions were: 10 min at 95°C, followed by 30 cycles of 10 s at 95°C, 1 min at 62°C, and 30 s at 72°C, followed by 1 min at 95°C, 30 s at 55°C, and 30 s at 95°C. In both reactions, the number of PCR cycles necessary to accumulate sufficient fluorescent signal to cross a threshold (Ct) was measured and individuals with relatively longer telomeres were characterized by shorter reaction times. All samples fell within the bounds of the standard curve and the reaction efficiencies were always within 100 ± 15%. Samples were run in triplicate, and mean values were used to calculate T/S ratios for each sample using the formula: 2^−ΔΔCt^, where ΔΔCt = (Ct^telomere^–Ct^GAPDH^)_sample_−(Ct^telomere^–Ct^GAPDH^)_reference_. Interplate repeatability of the T/S ratio was calculated for all samples (*n* = 32) run across two plates using the R package “rptr” (Stoffel et al., 2017), and the intraclass correlation coefficient was 0.94 ± 0.02 (95% CI: 0.87–0.97).

### 2.7 Statistical analysis

All statistical analyses were performed in *R* (version 3.6.3, RStudio Team, 2020) and are reported in the results as β‐estimate ± standard error. We performed a linear model (“stats” package) for each dependent variable: body mass, wing length, primary feather emergence length, and relative telomere length at 11 days old. We ran linear models with fixed effects of treatment, sex, a treatment by sex interaction, and clutch size. For the linear model predicting relative telomere length, we also included a fixed effect of body mass, which has been associated with telomere dynamics in previous work (Monaghan & Ozanne, 2018). For models with significant treatment by sex interactions, we performed post hoc comparisons using lsmeans (Lenth, 2016). We visually inspected all models for normal distribution of residuals, and nestling body mass was square transformed to achieve normality.

## 3 RESULTS

**FIGURE 2:**
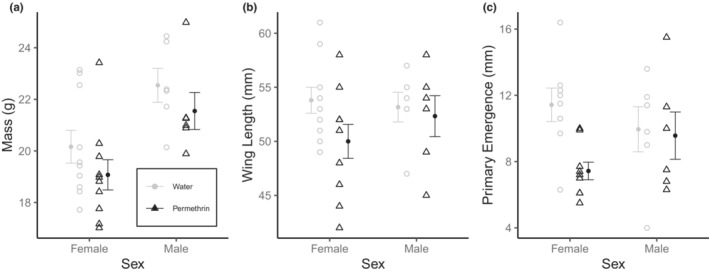
Morphological variation in 11‐day old tree swallow nestlings reared in nests treated with Permethrin (*n* = 10 females, 6 males) or water (*n* = 10 females, 6 males). Permethrin removal of ectoparasites did not affect body mass (a), but led to marginal decreases in wing length (b) and significant decreases in primary feather emergence (c). Means ± SE are shown.

Treatment of nests with permethrin had no effect on nestling body mass (β = −47.36 ± 36.10 g^2^, *F*_1,27_ = 2.41, *p* = .13), but males were larger than females (β = 96.52 ± 41.35 g^2^, *F*_1,27_ = 12.04, *p* = .002, Figure 2a). Clutch size (β = −10.24 ± 18.20, *F*_1,27_ = 0.31, *p* = .58) and the treatment by sex interaction (β = 3.81 ± 58.17, *F*_1,27_ = 0.004, *p* = .95) were nonsignificant. In addition, nestlings from permethrin‐treated nests had marginally shorter wings (β = −4.09 ± 1.98 mm, *F*_1,27_ = 3.08, *p* = .09, Figure 2b) with no effects of sex (β = −0.90 ± 2.27 mm, *F*_1,27_ = 0.29, *p* = .59), clutch size (β = −0.72 ± 1.00 mm, *F*_1,27_ = 0.36, *p* = .55), or a treatment by sex interaction (β = 3.26 ± 3.19 mm, *F*_1,27_ = 1.04, *p* = .32). Similarly, the ectoparasite‐removal group had significantly reduced primary feather emergence at 12 days old (β = −4.04 ± 1.39 mm, *F*_1,24_ = 5.64, *p* = .03, Figure 2c), with no effects of sex (β = −1.53 ± 1.55 mm, *F*_1,24_ = 0.12, *p* = .73), clutch size (β = −0.18 ± 0.66 mm, *F*_1,24_ = 0.01, *p* = .91), or a treatment by sex interaction (β = 3.66 ± 2.15 mm, *F*_1,24_ = 2.90, *p* = .10).

**FIGURE 3:**
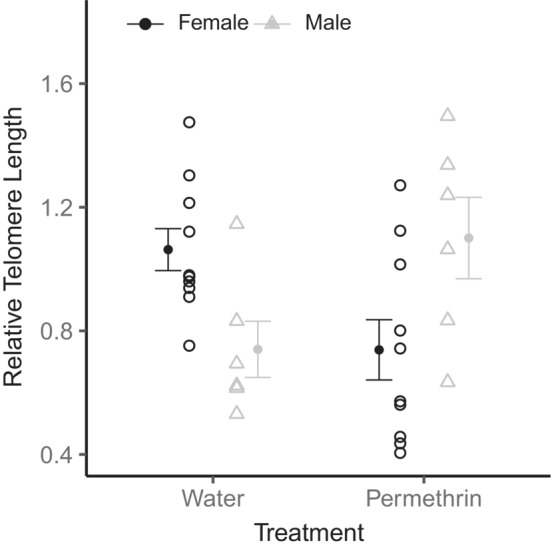
The effect of ectoparasite removal on blood relative telomere length quantified at 11‐day old. Data points are split by sex and treatment, in which nests were treated with Permethrin (*n* = 10 females, 6 males) or water (*n* = 10 females, 6 males). Males from permethrin‐treated nests have longer telomeres than controls; however, females did not differ across groups. Control females have longer telomeres than control males, but sexes do not differ in permethrin‐treated nests. Mean ± SE are shown.

There was a significant interaction effect of treatment and sex on nestling relative telomere length (β = 0.67 ± 0.19, *F*_1,26_ = 12.52, *p* = .002, Figure 3), with no main effects of treatment, sex, or clutch size (β = 0.03 ± 0.06, *F*_1,26_ = 0.36, *p* = .56). Heavier nestlings did have significantly longer telomeres (β = 0.05 ± 0.03, *F*_1,26_ = 5.31, *p* = .03). Post hoc comparisons of the treatment by sex interaction show that males raised in permethrin‐treated nests had significantly longer relative telomere lengths compared to control males (β = −0.42 ± 0.15, *t* = −2.78, *p* = .046). However, females in control and treated nests did not differ in telomere length (β = 0.25 ± 0.12, *t* = 2.06, *p* = .19). Consequently, control females had significantly longer telomeres than control males (β = 0.45 ± 0.15, *t* = 3.07, *p* = .02), but sexes did not differ in telomere length within permethrin‐treated nests (β = −0.21 ± 0.15, *t* = −1.47, *p* = .47).

## 4 DISCUSSION

Parasitism may exert selection pressure via effects on telomere length (e.g., Asghar et al., 2015; Ilmonen et al., 2008)—which often predicts vertebrate fitness (Wang et al., 2018; Whittemore et al., 2019; Wilbourn et al., 2018). Using a broad‐spectrum insecticide, we experimentally reduced blow fly (and possibly other ectoparasite) presence in the nests of tree swallows to assess sex‐specific effects on nestling telomere length. Ectoparasite removal resulted in longer telomere length in 11‐day old male nestlings relative to males in control nests, suggesting that the effects of blow fly presence are sex‐biased. Nestlings in treated nests also displayed slower wing development regardless of sex, which may reflect a relaxed pressure to develop and fledge quickly in nests from which parasites were removed (Martin et al., 2011; Saino et al., 1998). These effects of parasitism on telomeres may be particularly salient early in life when innate and adaptive immunity are developing (Aastrup & Hegemann, 2021; Palacios et al., 2009), and critically, may prime adult success (Benetos et al., 2013; Heidinger et al., 2012). Given that telomere length can predict longevity in many species, including tree swallows (Haussmann et al., 2005; Heidinger et al., 2012; Wilbourn et al., 2018), robust sex‐biased telomere dynamics in response to environmental stressors like parasites could disrupt sex ratios and influence population growth and persistence.

Experimental removal of blow flies induced sex‐specific effects on telomere length. Corroborating previous work on malarial parasites, release from parasitism yielded longer telomeres (as in Asghar et al., 2015; Asghar et al., 2016; Karell et al., 2017); however, this effect was limited to males. Similar male‐biased responses of telomere length to parasitism have been reported (Ilmonen et al., 2008; Sudyka et al., 2019) but are not universal (Tschirren et al., 2021), and such differences may stem from species‐ and sex‐specific physiology of both hosts and their parasites. Male nestlings may be more susceptible to parasitism than females, as shown in some vertebrates (Gorrell & Schulte‐Hostedde, 2008; Harrison et al., 2010; Klein, 2004; Moore & Wilson, 2002). This could be driven in part by larger, more easily targeted, and resource‐rich bodies of males (as shown in Figure 1a; Christe et al., 2003; Hawlena et al., 2005), as well as higher levels of circulating testosterone, although support is mixed (Foo et al., 2017; Roberts et al., 2004). Evidence of male susceptibility can be seen in Figure 2, in which males have shorter telomeres than females in parasitized nests, a pattern that disappears upon blow fly removal. While growing evidence shows that parasites shorten telomeres in mammals and birds (Ilmonen et al., 2008; Sudyka et al., 2019; Tschirren et al., 2021), untangling the factors determining the *directionality* of sex biases requires further studies using additional physiological responses and host–parasite pairs.

Immune activation by parasites may trade‐off with growth and reproduction (Graham et al., 2011; but see Aastrup & Hegemann, 2021; van der Most et al., 2011), and therefore, the removal of ectoparasites should increase nestling size. Although we saw no effects on nestling body mass, 11‐day‐old primary feather emergence, and wing length to a marginal but not statistically significant degree, were shorter in treated nests regardless of sex. Here, parasitism may favor accelerated growth to escape predation risk in the nest (Martin et al., 2011), and so it follows that nestlings from our permethrin‐treated nests exhibited slower growth in the absence of that pressure. Interestingly, if wing length at fledging (~21‐day old) became equal across treatments, the slower and longer wing growth of chicks from deparasitized nests may avoid costs of accelerated growth under parasitism and improve adult fitness (Metcalfe & Monaghan, 2001). Alternatively, less developed wings may impair flight and increase mortality rates after fledging the nest (Jones & Ward, 2020), e.g., when these aerial insectivores must acquire food, avoid predators, and migrate great distances within just a few months (Winkler et al., 2020). However, a nonmutually exclusive alternative is that the insecticide itself affected development. This was suggested by Bulgarella et al. (2020) and López‐Arrabé et al. (2014), in which nestling body mass and feather growth decreased in de‐parasitized nests treated with insecticide but not with heat (but see De Simone et al., 2018; Grab et al., 2019). However, if this insecticide proved toxic to nestlings, we would likely expect shorter telomeres—not longer, in permethrin‐treated nests.

Regardless, our study provides some support for sex‐specific effects of blow fly ectoparasitism on telomere length in nestling tree swallows. Male telomeres appeared more sensitive to the presence of *Protocalliphora* (and possibly other ectoparasites) than females. These findings contrast the female‐biased effect of hen fleas on telomeres shown in Tschirren et al. (2021), one of a few publications thus far that quantifies telomeres after direct manipulation of ectoparasites in birds. Critically, the directionality of sex biases in telomere dynamics is inconsistent across taxa (Remot et al., 2020). This makes studying the causes and consequences of telomere length at this young age—when telomeres change the most (Frenck et al., 1998; Spurgin et al., 2018), especially impactful for understanding sexual dimorphism in life‐history and aging.

## AUTHOR CONTRIBUTIONS

**Sarah Wolf:** Conceptualization (equal); formal analysis (lead); validation (lead); visualization (lead); writing – original draft (equal); writing – review and editing (equal). **Samuel Zhang:** Conceptualization (equal); investigation (supporting); writing – original draft (supporting); writing – review and editing (supporting). **Ethan D Clotfelter:** Conceptualization (equal); funding acquisition (lead); investigation (lead); project administration (lead); resources (lead); supervision (lead); writing – original draft (equal); writing – review and editing (equal).

## CONFLICT OF INTEREST STATEMENT

The authors declare no competing interests.

## Data Availability

Data are available from the Dryad Digital Repository: https://doi.org/10.5061/dryad.1zcrjdfws (Wolf et al., 2023).
